# Retinopathy in severe malaria in Ghanaian children - overlap between fundus changes in cerebral and non-cerebral malaria

**DOI:** 10.1186/1475-2875-9-232

**Published:** 2010-08-12

**Authors:** Vera A Essuman, Christine T Ntim-Amponsah, Birgitte S Astrup, George O Adjei, Jorgen AL Kurtzhals, Thomas A Ndanu, Bamenla Goka

**Affiliations:** 1 University of Ghana Medical School, College of Health Sciences-University of Ghana, Accra, Ghana; 2 Centre for Medical parasitology, Department of Clinical Microbiology, Copenhagen University Hospital, and Department of International Health, Immunology and Microbiology, University of Copenhagen, Denmark; 3 University of Ghana Dental School, College of Health Sciences-University of Ghana, Accra, Ghana

## Abstract

**Background:**

In malaria-endemic areas, reliably establishing parasitaemia for diagnosis of malaria can be difficult. A retinopathy with some features unique to severe malaria with a predictive value on prognosis, has been described. Detection of this retinopathy could be a useful diagnostic tool. This study was designed to determine the diagnostic usefulness of retinopathy on ophthalmoscopy in severe malaria syndromes: Cerebral malaria (CM) and non-cerebral severe malaria (non-CM), i.e. malaria with respiratory distress (RD) and malaria with severe anaemia (SA), in Ghanaian children. Secondly, to determine any association between retinopathy and the occurrence of convulsions in patients with CM.

**Methods and subjects:**

A cross-sectional study of consecutive patients on admission with severe malaria who were assessed for retinal signs, at the Department of Child Health, Korle-Bu Teaching Hospital, Accra, from July to August 2002 was done. All children had dilated-fundus examination by direct and indirect ophthalmoscopy.

**Results:**

Fifty-eight children aged between six months and nine years were recruited. Twenty six(45%) had CM, 22 with convulsion; 26(45%) had SA and six(10%) had RD.

Any retinopathy was seen in: CM 19(73%), SA 14(54%), RD 3(50.0%), CM with convulsion 15(68%) and CM without convulsion 4(100%). Comparison between CM versus non-CM groups showed a significant risk relationship between retinal whitening and CM(OR = 11.0, CI = 2.2- 56.1, p = 0.001). There was no significant association with papilloedema(OR = 0.9, CI = 0.3 - 3.0, p = 0.9), macular whitening(OR = 1.6, CI = 0.5 - 4.8, p = 0.4), macular haemorrhage(OR = 0.28, CI = 0.03 - 2.7 p = 0.2), retinal haemorrhage(OR = 1.9, CI = 0.6 - 5.6, p = 0.3), vessel abnormality(OR = 1.9, CI = 0.6 - 6.1, p = 0.3) and cotton wool spots(OR not calculated, p = 0.08).

Tortuous and engorged retinal veins, not previously described as a feature of CM, was the most common vascular abnormality(15/58 = 26%) and was detected even in the absence of papilloedema.

**Conclusion:**

Retinal whitening, a sign suggestive of retinal ischaemia, was significantly more common in CM than in non-CM syndromes. However, the high prevalence of any retinopathy in the latter suggests that the brain and the retina may be suffering from ischaemia in both CM and non-CM.

## Background

Malaria is an important cause of morbidity and mortality especially in sub-Saharan Africa [1], where children are most commonly affected [2,3]. The common malaria syndromes: cerebral malaria (CM), severe malarial anaemia (SA), and respiratory distress (RD), which is a sign of metabolic acidosis [3-5], account for the majority of malaria-related deaths in African children [4].

In malaria-endemic areas, reliably establishing parasitaemia for diagnosis and differentiating between malaria and other causes of febrile disease can be difficult [6], often complicated by the high prevalence of asymptomatic malaria which makes it possible for other febrile illnesses to be superimposed on the parasitaemia. This difficulty is further enhanced by the considerable overlap that exist between the clinical signs of severe malaria and other serious infectious diseases such as pneumonia [7], and other causes of coma [7-9], leading to a failure to treat some of these life-threatening diseases [6]. The need to improve on the diagnosis of severe malaria has led to the search for more reliable tools for establishing a diagnosis [10] in order to improve on the appropriate and timely use of interventions aimed at reducing the persistently high mortality of severe malaria [11].

A combination of retinal abnormalities have been described to be associated with severe malaria. Retinal changes were described more than 50 years ago in cerebral malaria [12]. Later macular lesions and signs of impaired circulation were also described [13,14]. Recently retinal changes have received renewed attention leading to important progress in the diagnosis, determination of prognosis [15-17] and the understanding of the pathogenesis of cerebral malaria [18-21] due to the extensive and detailed study done by the Malawi group. This included blood flow studies, histopathology and clinical aspects.

Characteristic features of these retinal changes include whitening of the peripheral retina and macula (sparing the central fovea), retinal vessel abnormalities, papilloedema, and multiple retinal haemorrhages often with pale centres [15,16,22-24]. The retinal vessel abnormalities are described as discoloration of retinal vessels to orange or white, mainly in the peripheral fundus. Either discrete sections of vessels, or peripheral trees, can be involved. White or orange tram lining within larger vessels may also be continuous or interrupted, delineating an apparently narrowed blood columns. Capillary whitening refers to whitening of retinal capillaries and post-capillary venules such that they become prominent against the choroidal background. This pattern of retinal whitening and the vessel changes appear to be unique to severe malaria and are not seen in any other ocular or systemic conditions [16,22], whereas the other findings can be seen in other conditions.

The detection of malarial retinopathy (especially retinal whitening and vascular changes) could therefore be a useful diagnostic tool for the clinician to confirm the diagnosis of severe malaria, particularly in a comatose parasitaemic child. The severity of retinopathy (number of retinal haemorrhages) and papilloedema may also be useful in predicting the likelihood of death in cerebral malaria [11,25]. Specifically, it has been suggested that ophthalmoscopy may be the most specific diagnostic tool, calling for widespread use in general practice [15].

In Ghana, malaria is endemic and a leading cause of death in children [26,27]. Improvement in the diagnosis of severe malaria, especially CM, and its differentiation from other causes of childhood coma might contribute to improved case management and possibly a reduction in mortality. Prior to initiating widespread training of clinicians in ophthalmoscopy for suspected CM, as suggested [15], the authors aimed to confirm the diagnostic usefulness of retinopathy on ophthalmoscopy in diagnosing the most common syndromes of severe malaria, i.e. CM versus non-CM (SA and RD) in Ghanaian children. As a secondary objective, the study aimed to determine the association between retinopathy and the occurrence of convulsions in patients with CM.

## Methods

### Patients

A cross-sectional study of consecutive patients on admission with severe malaria who were assessed for retinal signs, at the Department of Child Health, Korle-Bu Teaching Hospital, Accra, from July to August 2002 was carried out. Ethical approval for the study was obtained from the University of Ghana Medical School Ethics and Protocol Review Committee, and participation was dependent upon signed informed consent from the parents or guardian of the patients.

Children aged six months to nine years, presenting with signs and symptoms suggestive of malaria, axillary temperature ≥37.5°C and no other obvious cause of fever were consecutively screened for inclusion by a project physician.

Inclusion criteria were: Patients with *P. falciparum *parasitaemia who fell into one of the following categories of severe malaria: SA: haemoglobin (Hb) < 5 g/dL, fully conscious with no episodes of severe bleeding, and no reported or observed convulsions; CM: Blantyre coma score ≤ 3 and duration of coma > 60 minutes despite correction of any hypoglycaemia, any Hb value and no record of recent severe head trauma, no other cause of coma or neurological diseases; RD: rapid breathing plus one or more of the following: alar flare, chest recessions, use of accessory muscles for respiration, or abnormally deep breathing. Patients with evidence of other infectious disease, abnormal cerebrospinal fluid and positive sickling status were excluded. Clinical parameters were documented on standard case record forms.

### Ophthalmological investigation

All children were examined once by an ophthalmologist within 12 hours of admission, by direct and indirect ophthalmoscopy after pupil dilatation with tropicamide 1% and phenylephrine 2.5%. The retinal findings were recorded on predesigned standardized charts specifically including the previously reported findings associated with CM. Ophthalmoscopy was repeated for all patients at one week, and those who had persistent abnormalities were re-examined after one month. The eye findings used in the analysis for a child were the changes documented during admission.

### Diagnostic procedures and case management

Haemoglobin was measured using an 18-parameter, automatic haematology analyzer (Sysmex KX-21, Japan); screening for sickling using the sodium metabisulphite method; microscopic detection and identification of *Plasmodium *parasites using Giemsa-stained thick and thin blood films. Cerebrospinal fluid examination was done in patients with altered consciousness as soon as it was considered clinically safe in order to rule out meningitis.

As per institutional practice at the time, patients with CM and those with other forms of severe malaria who could not retain oral medication were treated with intramuscular quinine sulphate (10 mg/kg body weight, 8-hourly). IM quinine was changed to syrup quinine at the same dosage when patients regained full consciousness or after 72 hours (whichever was earlier), via nasogastric tube to complete a 7-day course. Patients with other forms of severe malaria who were able to take medications orally were treated with amodiaquine 10 mg/kg body weight per day, as single daily doses for three days. Patients with SA or RD were given humidified oxygen and children with SA were transfused with blood. Hypoglycaemia was corrected with intravenous 10% dextrose solution. Paracetamol and anticonvulsant therapy were administered as clinically indicated at standard dosage.

### Statistical analysis

Data was analysed using SPSS Version 16.0. Continuous numerical variables were summarised as mean and standard deviation (SD) and categorical variables as percentages with binomial confidence intervals. Group comparisons were done using Chi-square test (with Yate's correction for 2 × 2 comparisons) or Fischer's exact test as appropriate. Binary logistic regression was used for analysis of associations between retinal signs and level of haemoglobin. P-values < 0.05 were considered statistically significant.

## Results

Fifty-eight children aged between six months and nine years were recruited. Twenty-nine (50%) were males. The types of severe malaria encountered were: CM 26 (45%), SA 26 (45%) and RD 6 (10%). Twenty-two (85%) of the patients with CM had at least one convulsion either observed during admission or reported by parents or guardians or both. Nineteen (73%) patients with CM, 14 (54%) with SA and three (50.0%) with RD i.e. a total of 36 out of the 58 patients (62%), showed one or more retinal abnormalities (Table 1).

**Table 1:** Retinopathy in different types of Severe Malaria

	CM (n = 26)	SA (n = 26)	RD (n = 6)	CM with convulsion (n = 22)	CM without convulsion (n = 4)
Any retinophaty	19 (73%)	14 (54%)	3 (50%)	15 (68%)	4 (100%)
Retinal whitening	11 (42%)	2 (8%)	0 (0%)	8 (36%)	3 (75%)
Cotton wool spots	3 (12%)	0 (0%)	0 (0%)	3 (14%)	0 (0%)
Papilloedema	6 (23%)	6 (23%)	0 (0%)	5 (23%)	1 (25%)
Macular whitening	10 (39%)	7 (27%)	1 (17%)	9 (41%)	1 (25%)
Macular haemorrhage	1 (4%)	2 (8%)	0 (0%)	1 (5%)	0 (0%)
Retinal haemorrhage	11 (42%)	7 (27%)	2 (33%)	10 (46%)	1 (25%)
Vessel abnormalities	9 (35%)	6 (22%)	1 (17%)	6 (27%)	3 (75%)

CM = Cerebral Malaria, SA = Malaria with severe anaemia, RD = Malaria with respiratory distress

The retinopathies seen in this study included: retinal whitening (mid- and peripheral retina) 13 (22%); macular whitening 19 (33%); macular haemorrhages 5 (9%) and retinal haemorrhages (round shaped, some with white centres, and some flame-shaped) 20 (35%); papilloedema 12 (21%); cotton wool spots 3 (5%), and vessel abnormalities 16 (28%). The vessel abnormalities observed included whitening of the vessels especially at the periphery of the retina and white tram lining of vessels. However, orange discoloration of vessels was not encountered.

The commonest vascular abnormality was engorgement and tortuosity of veins (15/58 = 26%). These vessels extended from the periphery to the optic disc and 8 out of 58 (14%) were not associated with papilloedema.

In order to assess the clinical usefulness of retinopathy on ophthalmoscopy in differentiating between CM and non-cerebral severe malaria, i.e. the pooled group of SA and RD patients, the frequency of occurrence of retinopathy in the two groups was compared (Table 2). There was no statistically significant difference between the finding of any retinopathy and the clinical pattern.

**Table 2:** Odds ratio of retinopathy in cerebral malaria versus non-cerebral severe malaria

Condition	Odds ratio	95% CI	P-value
Any retinopathy	2.1	(0.7-6.4)	0.2
Retinal whitening	11.0	(2.2-56.1)	0.001
Cotton wool spots	NA	NA	0.084
Papilloedema	0.9	(0.3 - 3.0)	0.9
Macular whitening	1.6	(0.5 - 4.8)	0.4
Macular haemorrhage	0.3	(0.0-2.7)	0.2
Retinal haemorrhage	1.9	(0.6 - 5.6)	0.3
Retinal vascular abnormality	1.9	(0.6-6.1)	0.3

NA, not calculated (since there was zero frequency for the presence of Cotton wool spots in the non-CM group).

There was a significant risk relationship between retinal whitening and CM as compared with the non-CM (OR = 11.0, CI = 2.2- 56.1). It should be noted, however, that less than 50% of CM patients displayed these particular finding. A logistic binary regression analysis done to examine the effect of the level of haemoglobin and anaemia on the presence or absence of retinal whitening in the CM group failed to show any significant influence (OR = 0.9, CI = 0.1- 4.1, p = 0.6) (Data not shown). There were no significant risk relationships seen between CM and the rest of the retinal signs studied.

Fifteen (68%) of the CM patients with convulsions and 4(100%) of the CM patients without convulsions showed one or more retinal signs (Table 1). Two patients in the CM group had transient neurological deficits with subsequent full recovery by day 14. One of these, who had CM without convulsion had a right upper motor neurone facial nerve palsy and also showed macular and peripheral retinal whitening, as well as vascular abnormalities. The other, who had CM with convulsion, had ataxia and retinal haemorrhages with vascular abnormalities.

One week after admission and onset of treatment, all the initial retinal changes had disappeared in 23 out of 36 (61%) who had some retinopathy initially, i.e. the changes were reversible and none of the patients had developed new retinopathies. In particular, the engorged and tortuous vessels had disappeared by one week in 13 out of the 15 children and in the remaining 2 children this finding had disappeared by the one-month follow-up.

## Discussion

This study demonstrated retinopathies in all types of severe malaria corroborating the findings from the Gambia, Kenya, Malawi and Mali [15,16,22,23]. Other reported retinal abnormalities such as orange discoloration of vessels were not seen. Interestingly, previously unreported changes of tortuous and engorged veins, extending from the periphery to the optic disc were encountered. Such changes are not commonly observed in the background population and might be associated with malaria. The study did not include a healthy control group, but during patient follow up these vascular changes disappeared in 13 out of 15 children by the one-week follow-up and in the rest by one month, thus supporting such an association. This finding of tortuous and engorged veins has not been highlighted in other studies. Tortuous retinal veins are findings encountered in ophthalmology in relation to diverse ocular and systemic aetiologies such as retinal vein occlusions, diabetic retinopathy, hyperviscosity syndromes, and papilloedema from increased intracranial pressure among others. Some of these result in permanent structural changes with visual impairment [28]. The current study showed these vascular changes to be transient, possibly suggesting a mechanical obstruction by parasites in either or both central and peripheral veins or metabolic cause such as metabolic steal by intravascular parasites or hypoxic stress. This requires further studies.

Retinal whitening was the only retinopathy that showed significant association with CM in this study. Retinal whitening, which has been suggested as an indicator of retinal ischaemia due to capillary non-perfusion [19] and its significant association with CM in this study corroborates the point that the brain together with the retina may suffer from ischaemia in CM, since both form part of the central nervous system and share common features in circulation [19]. Other studies have demonstrated other retinopathies to be significantly associated with CM: from Mali and Malawi papilloedema was demonstrated to have significant relationship with CM and was also associated with increased mortality. In the study from Malawi (n = 326) retinopathy in CM was associated with subsequent death (relative risk, 3.7; 95% CI = 1.6-8.5) and papilloedema conferred the highest risk (relative risk, 4.5; 95% CI = 2.7-7.6) [16]. The study from Mali found the presence of exudates, papilloedema, and cotton-wool spots to be associated with an increased risk of death but no association was found between ocular signs such as retinal haemorrhages or retinal oedema and mortality [22]. However, in Ibadan, Nigeria, a study of 73 patients with CM found retinal haemorrhage to be significantly associated with death whereas papilloedema alone was not [25]. It is possible that these differences could be due to the diagnostic procedures or to differences in the background population e.g. nutritional status including Vitamin A [29] and may also be due to the linkage between the intensity of transmission of malaria in different epidemiological settings to the pattern of clinical presentation and age of onset in children [30], among other factors.

Of interest, however, is that this current study detected similar proportions of the various retinopathies as found in older studies from Malawi [31,32]. This suggests that once skill is acquired with the indirect ophthalmoscope the features of this retinopathy are clearly evident, and their diagnostic and prognostic value can be utilised in the diagnosis of severe malaria, especially CM, and its differentiation from other causes of childhood coma. It is the technique and equipment of indirect ophthalmoscopy, which remain the barriers to wider uptake and usefulness. Training in indirect ophthalmoscopy would thus be required to improve on uptake and usefulness. However, the high prevalence of any retinopathy in the non-cerebral severe malaria group limits the use of malarial retinopathy in differentiating CM from other severe malaria syndromes such as SA and RD. This low specificity has also been reported previously [16].

No deaths were encountered in this study and so the associations between retinal changes and deaths could not be studied. Other studies have demonstrated increased mortality and a positive predictive value of some malarial retinopathies (retinal whitening, papilloedema, and retinal haemorrhages) for fatality and severe malaria especially CM [11,16,22,25].

It was not possible to determine if there was any association between convulsion and retinopathy in CM because of the small number of patients who had no convulsion.

The two patients with neurological sequelae (upper motor neurone facial nerve palsy and ataxia, both recognized neurological complications of severe malaria) [16] had retinal changes.

The limitations of this study are that: since all the patients studied survived, it was impossible to determine the prognostic significance of the retinopathies observed for mortality. The small number of patients analysed for each type of severe malaria and the lack of comparison with non-cerebral malaria comas or uncomplicated malaria also contributed further to the limitations. Finally, this study used bedside ophthalmoscopy to test the clinical relevance of retinopathy in patients with severe malaria without fundus photography. Thus detailed measurement and evaluation of the calibre of the blood vessels encountered could not be done reliably.

## Conclusion

This study demonstrated a high occurrence (50-75%) of retinal changes in the patients with severe malaria (CM, SA, RD). Whereas detection of malarial retinopathy through ophthalmoscopy may potentially be very useful from a diagnostic, prognostic, and scientific point of view, the high prevalence of any retinopathy in the non-cerebral severe malaria group limits the use of malarial retinopathy in differentiating CM from other severe malaria syndromes.

Retinal whitening, a sign suggestive of retinal ischaemia, was found in both CM and non-CM syndromes, suggesting that the brain suffers from ischaemia in CM just as the retina, both forming part of the central nervous system.

Finally, once skill is acquired with the indirect ophthalmoscope the features of retinopathy are clearly evident and their diagnostic and prognostic value can then be utilised. It is the technique and equipment of indirect ophthalmoscopy which remain the barriers to wider uptake and usefulness.

## Competing interests

The authors declare that they have no competing interests.

## Authors' contributions

EV participated in the design of the study, performed the ocular examination and write up of the manuscript. NCT conceived and participated in the design of the study, performed the ocular examination and helped to draft the manuscript. AB was responsible for coordination of study. AGO and GBQ were responsible for patient selection and helped to prepare manuscript. NTA performed the statistical analysis and interpreted the data. KJAL conceived and participated in design of the study and helped to draft the manuscript.

All authors read and approved the final manuscript.
